# A Search History-Driven Offspring Generation Method for the Real-Coded Genetic Algorithm

**DOI:** 10.1155/2020/8835852

**Published:** 2020-09-27

**Authors:** Takumi Nakane, Xuequan Lu, Chao Zhang

**Affiliations:** ^1^University of Fukui, Fukui, Japan; ^2^Deakin University, Melbourne, Victoria, Australia

## Abstract

In evolutionary algorithms, genetic operators iteratively generate new offspring which constitute a potentially valuable set of search history. To boost the performance of offspring generation in the real-coded genetic algorithm (RCGA), in this paper, we propose to exploit the search history cached so far in an online style during the iteration. Specifically, survivor individuals over the past few generations are collected and stored in the archive to form the search history. We introduce a simple yet effective crossover model driven by the search history (abbreviated as SHX). In particular, the search history is clustered, and each cluster is assigned a score for SHX. In essence, the proposed SHX is a data-driven method which exploits the search history to perform offspring selection after the offspring generation. Since no additional fitness evaluations are needed, SHX is favorable for the tasks with limited budget or expensive fitness evaluations. We experimentally verify the effectiveness of SHX over 15 benchmark functions. Quantitative results show that our SHX can significantly enhance the performance of RCGA, in terms of both accuracy and convergence speed. Also, the induced additional runtime is negligible compared to the total processing time.

## 1. Introduction

Evolutionary algorithms (EAs) have been shown to be generic and effective to search for global optima in the complex search space theoretically [1–3] and practically [4–6]. The exploration process of EAs imitates the natural selection process, which is realized by conducting the offspring generation and survivor individual selection alternately and iteratively. The population quality is gradually improved throughout the exploration process, which can be viewed as a stochastic population-based generation-and-test process. Because of the offspring generation, a large number of candidate solutions (i.e., individuals) are sampled, accompanied by corresponding fitness values, genetic information, and genealogy information. Such accumulated search data constitute search history which can be very informative and valuable for boosting the overall performance. For instance, exploiting search history can be useful for improving the search procedure under a limited budget of fitness evaluations (FEs). That is, no additional FEs are allowed for improving the search performance. Also, the computational cost of a single FE can be high when the fitness functions are complicated. To enable a better solution for the population without increasing the number of FEs, the way of exploiting the search history truly matters. Nevertheless, search history has been sparsely exploited and studied in existing methods.

Real-coded genetic algorithm (RCGA) has been widely studied in the past decades [7–11], and the main efforts for improving the performance of RCGA have been focused on the development of the crossover techniques [12]. Because the crossover operator is to generate new offspring from the current population, the quality of the new solutions straightforwardly affects the evolution direction and convergence speed. Given different mechanisms, crossover methods can differ from (1) parent selection, (2) offspring generation, and (3) offspring selection. Both parent and offspring can be more than two, depending on the design. The abovementioned three aspects associate the exploration ability with exploitation ability, and the degree and balance between both abilities affect the performance largely [13]. Although the self-adaptive feature of RCGA [14] can adjust the relationship to a certain extent, the “best” degrees and balance between exploration and exploitation for achieving a satisfactory solution can differ greatly with respect to different problem settings and can be hardly achieved with the adaptive feature.

With a large amount of search history data up to the current generation in hand, we attempt to introduce a crossover method that effectively exploits the history data in this paper. At first, an archive is defined to collect the survivor individuals over generations as the search history. Then, the stored individuals are clustered by *k*-means [15], and each cluster is assigned a score depending on the number of belonging individuals. At last, offspring is generated and selected according to the scores. We introduce two different schemes to update the archive. The proposed crossover operator, named search history-driven crossover (SHX), generates offspring by considering the cluster scores. Since SHX enables an offspring selection mechanism, any existing parent selection and offspring generation mechanisms can be easily integrated with it. To our knowledge, this is the first work to design the crossover model by effectively exploiting search history. We present a set of experiments to systemically evaluate the effectiveness of the proposed method using 15 benchmark functions. Three conventional crossover operators are employed, and the results with/without SHX are compared. Apart from the above, two archive update methods are also analyzed.

The main technical contributions of this paper are threefold. First, we propose a novel crossover model by effectively exploiting the search history. Second, we introduce the offspring selection based on the clusters calculated from the search history. Third, we introduce two schemes to update the survivor archive. A preliminary version of this paper appears in GECCO2020 [16].

## 2. Related Work

Crossover is one of the principal operators for generating offspring and deeply relates to the performance of the real-coded genetic algorithm (RCGA). Blend-*α* crossover (BLX-*α*) [17] proposed by Eshelman and Schaffer is one of the most popular operators. Offspring genes are independently and uniformly sampled within an interval between a gene pair of parents. The parameter *α* corresponds to the extension of the sampling interval, which plays a key role in maintaining the diversity of offspring. Eshelman et al. proposed Blend-*α*-*β* crossover (BLX-*α*-*β*) [18] which involves two extension parameters. Deb and Agrawal introduced simulated binary crossover (SBX) [19] which simulates the single-point crossover in binary-coded GA for continuous search space. The interval used in SBX is determined by a polynomial probability distribution *β* depending on the distribution index *η*. *η* indirectly adjusts the tendency of offspring generation. The above crossover operators have a common feature that the offspring genes are extracted according to a certain probability distribution from the predefined interval on the parent genes. This feature enables better results than using crossover operators for binary coding in the continuous search space. On the other hand, some crossover operators set more than two individuals as parents, which aim to generate offspring with well-preserved population statistics. In the case of unimodal normal distribution crossover (UNDX) [20], the generation of offspring follows a unimodal normal distribution defined on the line connecting two of the three parents. For simplex crossover (SPX) [21], *D*+1 individuals are taken as parents in the *D*-dimensional search space. SPX uniformly generates offspring within *D*-dimensional simplex constructed by parent individuals and expanded by a parameter *ε*.

Search history has also been exploited in some research, but to the best of our knowledge, none of them is for the purpose of improving the crossover model. Since online real systems often provide uncertain evaluation values which lead to unreliable convergence of GA, Sano and Kita proposed memory-based fitness estimation GA (MFEGA) [22]. MFEGA estimates the fitness from neighboring individuals stored in the search history. Leveraging search history allows estimation without requiring additional evaluation. Amor and Rettinger proposed GA using self-organizing maps (GASOM) [23]. SOM (self-organizing maps) can provide a visualized search history, which makes the regions explored intuitive for users. Moreover, individual novelty is introduced by the activation frequency in the search history table and utilized by the reseeding operator to preserve the exploration power. Yuen and Chow presented the continuous nonrevisiting GA (cNrGA) [24]. A binary partitioning tree called a density tree stores all evaluated individuals and divides the search space into nonoverlapped partitions by means of distributions. These subregions are used to check whether a new individual needs to be evaluated or not.

## 3. Overview

**Figure 1:**
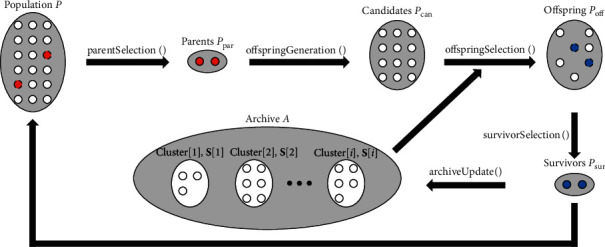
Overview of the proposed method. The proposed method is performed with an archive **A** under the framework of RCGA. **A** preserves survivors **P**_sur_ over the past few generations and extracts statistics from them by clustering. Offspring **P**_off_ are selected from excessively generated candidate solutions **P**_can_ based on the statistics.

Principles of designing good crossover operators for RCGA are discussed in [25]. Two among them are especially important: (1) the crossover operator should preserve the statistics of the population; (2) the crossover operator should generate offspring with as much diversity as possible under the constraint of (1). By following these suggestions, the key idea of SHX is to cluster the search history and select population members from excessively generated candidate solutions by preserving the statistics represented by the clusters.  Figure 1 illustrates the overview of our SHX. The proposed method is performed under the framework of RCGA which mainly involves survivor selection and crossover. Mutation is optional, but we exclude it to clearly investigate the effectiveness of SHX in this work.

The proposed method is described in Algorithm 1. Population is denoted by **P** which comprises *n*_*P*_ individuals, and the population at the *t*-th generation is denoted as **P**^*t*^. Similarly, parents for SHX, excessively generated candidate solutions during SHX, offspring after SHX, and survivors for the next generation are represented by **P**_par_, **P**_can_, **P**_off_, and **P**_sur_, respectively. The size of each set is denoted using *n* with a subscript of the set name (e.g., the size of parents is denoted by *n*_*P*_par__). In addition to **P**, our method manages an archive **A** which preserves *n*_*A*_ survivors throughout the generation alternation. **A** and **P** are initialized by randomly placing individuals in the search space. The archive update process is conducted after the survivor selection. Survivor individuals **P**_sur_ of the current generation are aggregated into both **P** and **A** of the next generation. SHX can be further divided into parent selection, offspring generation, and offspring selection. Different from conventional RCGA, individuals generated from **P**_par_ are regarded as offspring candidates **P**_can_. The main purpose of SHX is to narrow down **P**_can_ to *n*_*P*_off__ individuals denoted by **P**_off_ according to the statistics provided by **S**. **S** is calculated from the clustering result of the archive and immediately impacts the offspring selection.

SHX can adopt any existing crossover operators (e.g., BLX-*α* [17] and SPX [21]) for the offspringGeneration function (Algorithm 1, line 8) to generate **P**_can_ from **P**_par_. For the parentSelection function (Algorithm 1, line 6) and the survivorSelection function (Algorithm 1, line 11), the just generation gap (JGG) [26, 27] is employed in this work. That is, the parentSelection function randomly extracts *n*_*P*_par__ individuals from **P** as **P**_par_, and the survivorSelection function selects top-*n*_*P*_sur__ individuals in **P**_off_ as **P**_sur_ according to the fitness value. To show the performance increase brought by SHX, we choose the widely applied BLX-*α*, SPX, and UNDX for the offspring generation and compare the results in Section 6. We explain archiveUpdate (Algorithm 1, lines 3 and 13) and offspringSelection (Algorithm 1, line 9) in detail in Section 4 and Section 5, respectively.

## 4. Survivor Archive

Since the genetic operations are run alternately and iteratively, collecting and analyzing the history data may be beneficial for boosting performance. Given that SHX is to maintain the historical statistics **S** while producing offspring for the next generation, the archive **A** is designed to store **P**_sur_ over few past generations and extracts statistics **S**. The calculation of **S** is based on the *k*-means, which is an off-the-shelf nonsupervised clustering method. The pseudocode of *k*-means is shown in Algorithm 2. In particular, *k*-means is employed to cluster the individuals in **A** based on their position in the search space, and **S** is a normalized frequency histogram to show the proportion of each cluster size to *n*_*A*_. A higher score indicates that the corresponding cluster is more likely to be a promising search region. The statistics can then be maintained by probabilistically assigning newly generated candidates to each cluster according to **S**.

To keep the computational cost brought by *k*-means within an acceptable and constant range, the archive size is fixed to *n*_*A*_. That is, a part of individuals in **A** must be replaced with new survivors **P**_sur_ during the archive update to incorporate new information. Two types of update methods are considered in this work: (1) randomly selecting individuals in **A** and replacing them with **P**_sur_ (denoted by random); (2) replacing a part of **A** with **P**_sur_ in the order in which the individuals of **A** arrived (denoted by sequential). The performance comparison between these two approaches is discussed in Section 6.

The update of **A** and calculation of **S** are executed in the function archiveUpdate (Algorithm 1, lines 3 and 13) which is summarized in Algorithm 3. At the replacement step (Algorithm 3, line 4), *n*_*P*_sur__ individuals are discarded from **A** based on random or sequential approaches, and new **P**_sur_ are stored to **A**. Initialization is executed when *t* equals 0. The *k*-meansFit function (Algorithm 3, line 7) updates the centroids of the clusters according to the updated **A** and assigns updated cluster labels to each individual in **A**. After that, the normalized frequency histogram **S** for each cluster is calculated by the hist function (Algorithm 3, line 9) for further usage in offspring selection (Algorithm 4). Note that the initial centroids of the clusters in the current generation are inherited from the previous generation, as most individuals in **A**^*t*^ are the same as **A**^*t*−1^.

## 5. Search History-Driven Crossover (SHX)

SHX randomly selects parents by following the strategy of existing crossover operators (e.g., two parents in the case of BLX and *D*+1 parents in the case of SPX) and excessively generates candidate offspring **P**_can_ for further offspring selection. *n*_*P*_can__ ≫ *n*_*P*_off__ because **P**_can_ must ensure a sufficient number of individuals that can be assigned to each cluster in **A**. Here, generating individuals excessively can also be considered as a mechanism of diversity preservation. It is worth pointing out that the offspring selection is a different procedure from the survivor selection. Offspring selection belongs to the crossover model and is conducted before fitness evaluation. Survivor selection is conducted after fitness evaluation. Offspring selection narrows down **P**_can_ to **P**_off_ based on roulette wheel selection [28]. Each proportion of the wheel relates to each possible selection (i.e., clusters), and **S** is used to associate a probability of selection with each cluster in **A**. This can also be viewed as a procedure that SHX preferentially selects individuals in more “promising” regions. This bias selection can encourage the evolution of the population and accelerate the whole convergence. Besides, the statistics of the population (e.g., cluster size) can be maintained between two consecutive generations because the new generation is sampled based on the statistics of the history. Also, the diversity of **P**_off_ can be preserved because each newly generated individual from **P**_can_ has a probability to be assigned to**A**.

The algorithm of offspring selection is shown in Algorithm 4. Input **P**_can_ is excessively generated by existing crossover operators (Algorithm 1, line 8). Each candidate is labeled by the *k*-means Predict function (Algorithm 4, line 1) based on the current clusters estimated from **A**. Then, the roulette is constructed based on **S**. The roulette selection is called *n*_*P*_off__ times, yielding *n*_*P*_off__ selected offspring. Each time of roulette selection produces a cluster ID, and one candidate in **P**_can_ that belongs to the corresponding cluster is randomly selected and assigned to **P**_off_. To avoid duplicate selection, a selected candidate will be excluded from **P**_can_. If no more candidates correspond to a certain cluster (this is rarely the case by assuming *n*_*P*_can__ ≫ *n*_*P*_off__), the roulette is reconstructed by eliminating the proportion of the corresponding cluster. Finally, **P**_off_ is passed to the survivor selection process which determines **P**_sur_ using JGG.

## 6. Experimental Results

The performance of SHX is investigated over 15 benchmark functions, with each function in two different dimension settings. We comprehensively compare the performance of RCGA with/without SHX, and SHX is run with different settings of archive update methods (random/sequential) and offspring generation methods (BLX [17]/SPX [21]/UNDX [20]).

### 6.1. Experimental Setup

Benchmark functions are a useful tool to verify the effectiveness of a method, and it is general to use several functions with different properties, such as in [29, 30]. We selected 15 benchmark functions with different characteristics from the literature [31–33] for evaluation. Detailed information of each function is summarized in Table 1. Initialization of the population and the archive is conducted within the range provided by the 4th column in Table 1. It is worth mentioning that the searching space (i.e., range of parameters) during the generation alternation is not constrained. Each function is labeled according to different combination of characteristics (U + S, U + NS, M + S, and M + NS). By involving various characteristics of functions, we can analyze the proposed method more comprehensively and objectively. Furthermore, as all selected functions are adjustable in the setting of dimension, we adopt two different numbers of dimensions (*D*=5 and *D*=10) to control the difficulty degree of the search problem.

The setting of hyperparameters of the proposed method is listed in Table 2. The proposed method includes hyperparameters of not only RCGA (number of generations, *n*_*P*_, and *n*_*P*_off__) but also SHX (*n*_*P*_can__, *n*_*A*_, and *k*). Basically, the search problem defined by each function becomes more hard as the number of dimensions increases, which requires a lot of evaluations. For adaptive adjustment, the number of generations, *n*_*P*_, and *n*_*P*_off__ are set proportional to the number of dimensions. The constant values of each parameter are empirically determined because the purpose of the experiments is to validate the effectiveness of having SHX, rather than achieving the best solution for each function.

All experiments are executed 100 times with different random seeds. In each experiment, the generation alternation completely executed the number of generation times defined in Table 2. For a fair comparison, iterations under the same random seed start using the same population. The runtime and fitness are recorded with Python implementation (without either parallelization or optimization) on a i7-7700 CPU at 3.60 GHz, 12.0 GB RAM desktop computer.

### 6.2. Comparison in the Final-Generation-Elite

The results of the absolute error between the optimal value and the final-generation-elite fitness with respect to all combinations of functions, dimension, and methods are displayed in Table 3. Table 3 shows the minimum, maximum, median, mean, standard deviation (SD), and *p* value of the Mann–Whitney *U* test by each combination. The Mann–Whitney *U* test evaluates the significance of SHX results against results without SHX under the significance level *p*=0.05. Before showing the superiority by involving SHX, we first exclude a few results that all the methods are trapped by local optima or cannot reach the global optima. (1) *Easom Functionf*_8_. This function has several local minima. It is unimodal, and the global minimum only has a small area corresponding to the search space, which can be hardly arrived at. (2) *Schwefel 2.26f*_10_. Since the setup of this experiment does not restrict the range of parameters during search, an extremely small fitness value (even smaller than the global optimum) can be achieved with this function, which is not suitable for comparisons.

From Table 3, we can observe the clear improvement of performance brought by SHX. The results of the *p* value show that the methods with SHX have recognized the significance at least in 23 settings among all 30 settings. In the other five results (minimum, maximum, median, mean, and SD), the methods without SHX cannot achieve outperformed results for most settings. For instance, focusing on the minimum results, the methods without SHX outperform the methods with SHX only 5, 0, and 4 times by BLX, SPX, and UNDX, respectively. On the other hand, SHX with sequential archive update achieves the best performance. SH-BLX_sequential, SH-SPX_sequential, and SH-UNDX_sequential show the significance in 27, 26, and 27 settings, respectively. In addition, they achieve the best results in most settings with respect to the maximum, median, and mean results. One possible reason for sequential outperforming random in most cases is that sequential removes the oldest individual which arrived first, and therefore SHX can select offspring according to the up-to-date search history to reflect the trend of evolution more sensitively. In contrast, random uniformly removes individuals in the archive, which may impede the discovery of new solutions since old individuals may be retained for more generations in the archive.

#### 6.2.1. Analysis on BLX vs. SH-BLX

It has been already known that the standard BLX [17] faces difficulties especially when the target function is nonseparable [34] due to the parameter-wise sampling. By observing the results of *f*_4_ to *f*_7_ and *f*_12_ to *f*_15_ from Table 3, we can find that involving SHX significantly improves the performance, which indicates that SHX can help BLX to greatly mitigate this drawback. It is easy to understand because offspring selection with clusters embeds distance measure which builds the relationship among parameters.

#### 6.2.2. Analysis on SPX vs. SH-SPX

SPX [21] is a better alternative of BLX, and we can observe from Table 3 that SPX noticeably outperforms BLX. From Table 3, it is also very clear that SHX further boosts the performance of SPX to a large extent. In particular, the results of minimum and median are improved by involving SHX for all settings. As pointed out in [21], SPX has the ability to maintain the mean and covariance of the parent individuals, which is consistent with the design guideline of good crossover operators mentioned in Section 3. Since SHX manages an archive that stores search history over few generations, it can preserve some useful statistics (e.g., centroids of clusters) much longer. That is why SHX is able to enhance SPX.

#### 6.2.3. Analysis on UNDX vs. SH-UNDX

Similar to BLX and SPX, Table 3 shows that the results involving SHX are improved in most settings. UNDX is also designed to generate offspring inheriting the distribution of the parent individuals [35]. Therefore, statistics of the search history provided by SHX are useful for UNDX to enhance search ability.

### 6.3. Comparison in Convergence Curve

**Figure 2:**
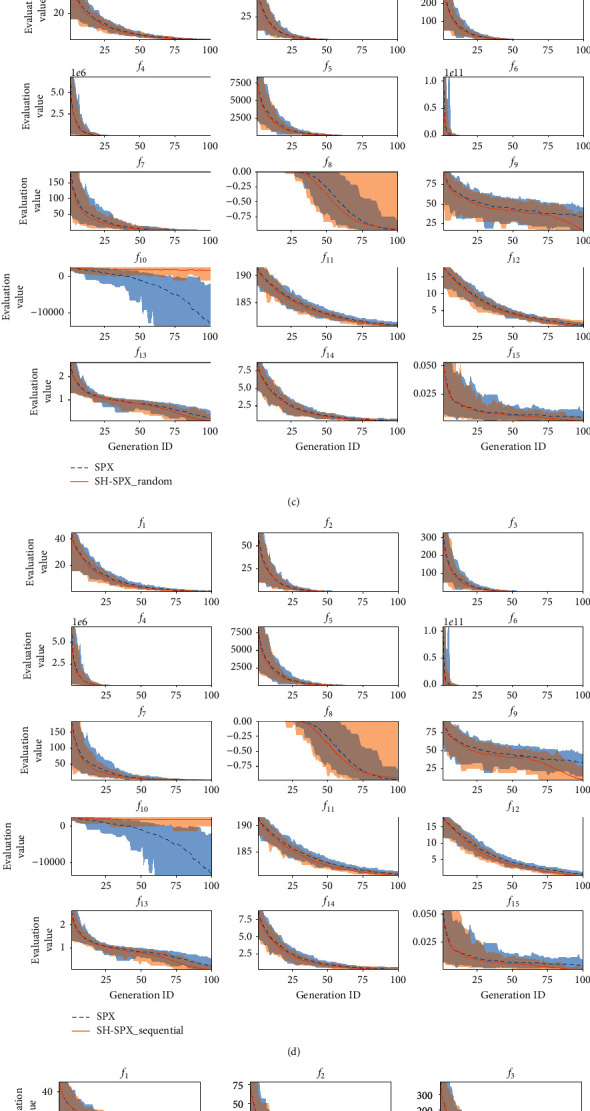
Convergence curves of all test functions. Each mean-min-max curve has a corresponding shaded area to represent the range of changes over 100 trials with different random seeds.

With the aid of search history, SHX not only achieves better results but also improves the convergence speed. In this section, we compare the generation alternation for over all the test functions in the case of *D*=10. Evaluation values of elite individuals from the 1st generation to the 100th generation are plotted in Figure 2. The mean value of 100 trials is represented by the line, and the range between the minimum and the maximum is represented by the shaded area. Smaller area means more stable search. It should be noted that as the ranges of parameters are not constrained during the search procedure, methods can achieve infinitely small values of fitness, and a lower value does not mean a better result in the case of *f*_10_, as explained in Section 6.2.

For BLX, SPX, and UNDX, exploiting SHX shows faster convergence speed comparing against them without SHX in most cases. The superiority becomes more obvious when the problem setting is more difficult (e.g., multimodal functions *f*_8−15_ vs. unimodal functions *f*_1−7_).

### 6.4. Comparison in Processing Time

**Figure 3:**
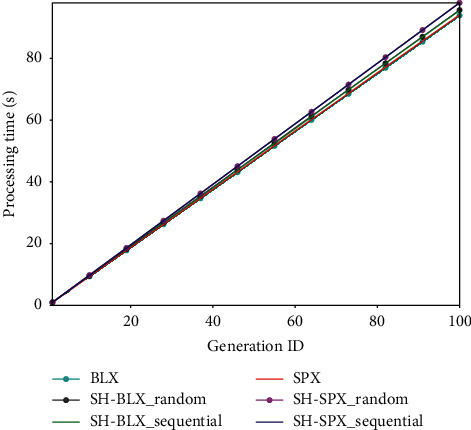
Processing time of different methods with increasing number of generations. The computational time is 0.01 second for a single evaluation.

In this section, we show the runtime overhead of the processing brought by SHX.  Figure 3 shows the comparisons in processing time of an optimization task (*D*=10 and a single fitness evaluation takes 0.01 second) for BLX and SPX. The parameter setting follows Table 2, and all the results are averaged over 10 trials. It took 93.9 seconds and 94.1 seconds for BLX and SPX to complete the entire process, respectively. SH-BLX_random took additional 1.7 seconds to BLX. SH-BLX_sequential took 1.6 seconds more than BLX. Similarly, the additional runtime for SH-SPX_random and SH-SPX_sequential to SPX were 3.9 seconds and 3.9 seconds, respectively. These numbers demonstrate the additional runtime only occupies a small part of the total processing time. These additional computational costs mainly occur in the clustering with archive data and the label assignment with candidate offspring. The cost can be further reduced by fusing efficient distance measure or parallel computing. For a fixed size of an archive, the runtime grows linearly with the increase in the number of generations. Considering the complexity of the fitness function and the budget, SHX is a practical alternative to other crossover models.

## 7. Conclusions

In this paper, we have proposed a novel crossover model (SHX) which is simple yet effective and efficient. It can be easily integrated with any existing crossover operators. The key idea is to exploit search history over generations to gain useful information for generating offspring. Experimental results demonstrate that our SHX can significantly boost the performance of existing crossovers, in terms of the final solution and the convergence speed. Also, according to experiments, the induced extra runtime is negligible compared to the total processing time.

SHX still has a few limitations. (1) Additional hyperparameters need to be determined. (2) The induced additional runtime may be unable to sufficiently support applications which require high processing speed. As the future work, we would like to address the above limitations. For instance, hyperparameters can be adaptively set by considering specific contexts, and parallelization can be introduced to speed up SHX.

## Figures and Tables

**Algorithm 1:**
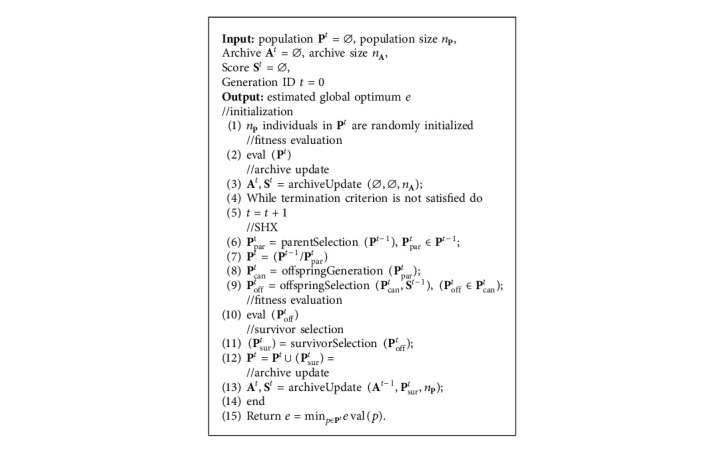
Search history-driven crossover for RCGA.

**Algorithm 2:**
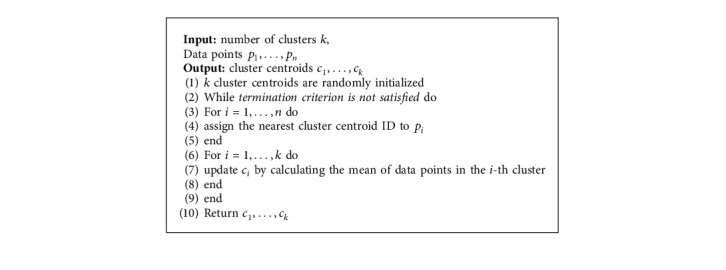
*k*-means.

**Algorithm 3:**
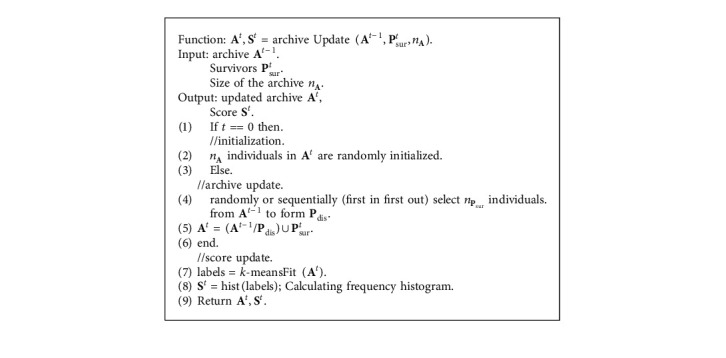
Archive update.

**Algorithm 4:**
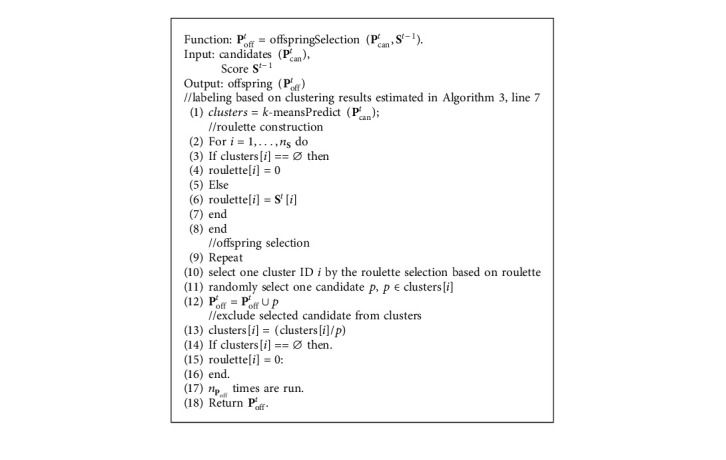
Offspring selection.

**Table 1:** Benchmark functions *f*_1_ ~ *f*_15_ used in the experiments.

ID	Name	Definition	Range	Label
*f*_1_	Schwefel 2.21	*f*(**x**)=max_1≤*i*≤*D*_|*x*_*i*_|	[−100,100]	U, S
*f*_2_	Sphere	*f*(**x**)=∑_*i*=1_^*D*^*x*_*i*_^2^	[−10,10]	U, S
*f*_3_	Sum squares	*f*(**x**)=∑_*i*=1_^*D*^*ix*_*i*_^2^	[−10,10]	U, S
*f*_4_	Rosenbrock	*f*(**x**)=∑_*i*=1_^*D*−1^[100(*x*_*i*+1_ − *x*_*i*_^2^)^2^+(*x*_*i*_ − 1)^2^]	[−30,30]	U, NS
*f*_5_	Schwefel 1.2	*f*(**x**)=∑_*i*=1_^*D*^(∑_*j*=1_^*i*^*x*_*j*_)^2^	[−100,100]	U, NS
*f*_6_	Schwefel 2.22	*f*(**x**)=∑_*i*=1_^*D*^|*x*_*i*_|+∏_*i*=1_^*D*^|*x*_*i*_|	[−100,100]	U, NS
*f*_7_	Zakharov	*f*(**x**)=∑_*i*=1_^*D*^*x*_*i*_^2^+((1/2)∑_*i*=1_^*D*^*ix*_*i*_^2^)^2^+((1/2)∑_*i*=1_^*D*^*ix*_*i*_^2^)^4^	[−5,10]	U, NS
*f*_8_	Easom	*f*(**x**)=(−1)^*D*^(∏_*i*=1_^*D*^cos^2^(*x*_*i*_))exp[−∑_*i*=1_^*D*^(*x*_*i*_ − *π*)^2^]	[−2*π*, 2*π*]	M, S
*f*_9_	Rastrigin	*f*(**x**)=10 *D*+∑_*i*=1_^*D*^(*x*_*i*_^2^ − 10 cos(2*πx*_*i*_))	[−5.12, 5.12]	M, S
*f*_10_	Schwefel 2.26	$f\left(x\right)=418.9829\;D-\sum_{i=1}^{D}x_{i}sin\left(\sqrt{\left|x_{i}\right|}\right)$	[−500,500]	M, S
*f*_11_	Weierstrass1	*f*(**x**)=∑_*i*=1_^*D*^[∑_*k*=0_^*k*max^*a*^*k*^cos(2*πb*^*k*^(*x*_*i*_+0.5)) − *D*∑_*k*=0_^*k*max^*a*^*k*^cos(*πb*^*k*^)]	[−0.5, 0.5]	M, S
*f*_12_	Ackley 1	$f\left(x\right)=-20\;\exp\left(-0.2\sqrt{\left(1/D\right)\sum_{i=1}^{D}x_{i}^{2}}\right)-\exp\left(\left(1/D\right)\sum_{i=1}^{D}\cos\left(2\pi x_{i}\right)\right)+20+\exp\left(1\right)$	[−35,35]	M, NS
*f*_13_	Griewank	$f\left(x\right)=\left(1/4000\right)\sum_{i=1}^{D}x_{i}^{2}-\prod_{i=1}^{D}\cos\left(x_{i}/\sqrt{i}\right)+1$	[−100,100]	M, NS
*f*_14_	Salomon	$f\left(x\right)=1-\cos\left(2\pi \sqrt{\sum_{i=1}^{D}x_{i}^{2}}\right)+0.1\sqrt{\sum_{i=1}^{D}x_{i}^{2}}$	[−100,100]	M, NS
*f*_15_	Xin-She Yang 2	*f*(**x**)=(∑_*i*=1_^*D*^|*x*_*i*_|)exp[−∑_*i*=1_^*D*^sin(*x*_*i*_^2^)]	[−2*π*, 2*π*]	M, NS

The last column (Label) represents the characteristics that the functions hold: unimodal (U), multimodal (M), separable (S), and nonseparable (NS).

**Table 2:** Hyperparameters of RCGA and SHX (*n*_*P*_off__, *n*_*P*_can__, *n*_*A*_,  and *k*).

Parameter	Value
Number of generations	10 *D*
Population size, *n*_*P*_	10 *D*
Number of offspring, *n*_*P*_off__	6 *D*
Number of candidates, *n*_*P*_can__	3*n*_*P*_off__
Archive size, *n*_*A*_	30*n*_*P*_sur__
Number of clusters, *k*	⌈*n*_**A**_/2⌉

*D* is the number of dimensions of test functions. All the parameters are fixed throughout the experiments.

**Table 3:** The results of the absolute error between the optimal value and the final-generation-elite fitness over 100 runs.

*f*	*D*	Minimum	Maximum Mean	Minimum	Maximum	Minimum	Maximum
		Median		Median	Mean	Median	Mean
		SD		SD	*p* value	SD	*p* value
		BLX		SH-BLX_random		SH-BLX_sequential	
*f*_1_	5	3.37*E* + 00	1.58*E* + 01	**2.07*E* + 00**	1.51*E* + 01	2.45*E* + 00	**1.36*E* + 01**
		8.82*E* + 00	8.92*E* + 00	7.27*E* + 00	**7.26*E* + 00**	**7.13*E* + 00**	7.35*E* + 00
		±2.87*E* + 00		±2.40*E* + 00	**2.76*E* − 05**	±**2.29*E* + 00**	**6.54*E* − 05**
	10	1.03*E* + 01	2.58*E* + 01	**8.60*E* + 00**	**2.28*E* + 01**	9.20*E* + 00	2.68*E* + 01
		1.80*E* + 01	1.84*E* + 01	1.72*E* + 01	**1.68*E* + 01**	**1.66*E* + 01**	1.69*E* + 01
		±3.20*E* + 00		±**2.90*E* + 00**	**1.11*E* − 03**	±3.36*E* + 00	**8.49*E* − 04**
*f*_2_	5	1.07*E* − 01	3.40*E* + 00	**1.19*E* − 02**	**1.69*E* + 00**	7.60*E* − 02	2.14*E* + 00
		7.75*E* − 01	9.28*E* − 01	5.67*E* − 01	**5.57*E* − 01**	**4.47*E* − 01**	5.63*E* − 01
		±6.39*E* − 01		±**3.78*E* − 01**	**3.07*E* − 06**	±4.11*E* − 01	**1.67*E* − 06**
	10	1.02*E* + 00	1.59*E* + 01	**9.51*E* − 01**	9.47*E* + 00	1.29*E* + 00	**8.85*E* + 00**
		5.02*E* + 00	5.33*E* + 00	4.14*E* + 00	4.24*E* + 00	**3.79*E* + 00**	**4.09*E* + 00**
		±2.18*E* + 00		±1.68*E* + 00	**8.78*E* − 05**	±**1.53*E* + 00**	**3.00*E* − 06**
*f*_3_	5	1.61*E* − 01	9.02*E* + 00	**1.51*E* − 01**	9.28*E* + 00	1.58*E* − 01	**5.74*E* + 00**
		2.08*E* + 00	2.62*E* + 00	**1.24*E* + 00**	**1.60*E* + 00**	1.35*E* + 00	1.69*E* + 00
		±1.66*E* + 00		±1.33*E* + 00	**6.50*E* − 08**	±**1.26*E* + 00**	**1.17*E* − 06**
	10	4.57*E* + 00	5.66*E* + 01	**2.41*E* + 00**	4.86*E* + 01	4.04*E* + 00	**4.56*E* + 01**
		2.47*E* + 01	2.60*E* + 01	**1.84*E* + 01**	2.11*E* + 01	2.02*E* + 01	**2.06*E* + 01**
		±1.02*E* + 01		±9.57*E* + 00	**1.33*E* − 04**	±**8.79*E* + 00**	**1.90*E* − 04**
*f*_4_	5	**6.96*E* + 01**	1.94*E* + 04	1.21*E* + 02	8.83*E* + 03	7.78*E* + 01	**8.08*E* + 03**
		1.94*E* + 03	2.80*E* + 03	8.98*E* + 02	1.74*E* + 03	**8.68*E* + 02**	**1.40*E* + 03**
		±2.80*E* + 03		±2.00*E* + 03	**7.07*E* − 05**	±**1.45*E* + 03**	**9.29*E* − 07**
	10	6.89*E* + 03	3.50*E* + 05	**4.44*E* + 03**	2.12*E* + 05	4.89*E* + 03	**1.56*E* + 05**
		6.11*E* + 04	7.19*E* + 04	4.69*E* + 04	5.35*E* + 04	**3.38*E* + 04**	**4.21*E* + 04**
		±5.19*E* + 04		±3.91*E* + 04	**1.73*E* − 03**	±**3.01*E* + 04**	**2.16*E* − 07**
*f*_5_	5	2.45*E* + 01	5.58*E* + 02	1.81*E* + 01	6.65*E* + 02	**7.61*E* + 00**	**4.54*E* + 02**
		1.78*E* + 02	1.93*E* + 02	1.30*E* + 02	1.63*E* + 02	**1.29*E* + 02**	**1.45*E* + 02**
		±1.15*E* + 02		±1.18*E* + 02	**9.22*E* − 03**	±**8.49*E* + 01**	**1.15*E* − 03**
	10	4.00*E* + 02	2.80*E* + 03	3.94*E* + 02	**2.29*E* + 03**	**2.98*E* + 02**	2.32*E* + 03
		1.40*E* + 03	1.37*E* + 03	**1.12*E* + 03**	**1.18*E* + 03**	1.16*E* + 03	1.22*E* + 03
		±4.28*E* + 02		±**3.99*E* + 02**	**6.21*E* − 04**	±4.39*E* + 02	**6.82*E* − 03**
*f*_6_	5	4.00*E* + 00	2.12*E* + 02	**2.06*E* + 00**	8.97*E* + 01	2.70*E* + 00	**8.71*E* + 01**
		2.52*E* + 01	3.36*E* + 01	**1.49*E* + 01**	2.05*E* + 01	1.56*E* + 01	**1.87*E* + 01**
		±3.25*E* + 01		±1.62*E* + 01	**4.48*E* − 06**	±**1.32*E* + 01**	**2.58*E* − 07**
	10	4.03*E* + 01	1.17*E* + 06	**1.63*E* + 01**	**1.57*E* + 05**	3.60*E* + 01	8.26*E* + 05
		3.85*E* + 03	3.75*E* + 04	**9.76*E* + 02**	**9.57*E* + 03**	1.09*E* + 03	1.31*E* + 04
		±1.33*E* + 05		±**2.77*E* + 04**	**5.04*E* − 05**	±8.24*E* + 04	**3.11*E* − 04**
*f*_7_	5	**2.84*E* − 01**	1.22*E* + 01	4.40*E* − 01	1.09*E* + 01	3.23*E* − 01	**1.02*E* + 01**
		3.33*E* + 00	3.87*E* + 00	2.48*E* + 00	2.93*E* + 00	**2.16*E* + 00**	**2.76*E* + 00**
		±2.52*E* + 00		±**1.97*E* + 00**	**3.21*E* − 03**	±1.99*E* + 00	**5.01*E* − 04**
	10	7.23*E* + 00	4.17*E* + 01	7.08*E* + 00	4.04*E* + 01	**5.92*E* + 00**	**4.00*E* + 01**
		2.39*E* + 01	2.32*E* + 01	1.98*E* + 01	2.04*E* + 01	**1.95*E* + 01**	**1.94*E* + 01**
		±7.22*E* + 00		±**6.47*E* + 00**	**1.49*E* − 03**	±6.75*E* + 00	**7.14*E* − 05**
*f*_8_	5	—	—	—	—	—	—
		—	—	—	—	—	—
		—		—	—	—	—
	10	8.70*E* − 01	1.00*E* + 00	**4.90*E* − 01**	**1.00*E* + 00**	9.16*E* − 01	1.00*E* + 00
		1.00*E* + 00	9.95*E* − 01	1.00*E* + 00	**9.86*E* − 01**	**1.00*E* + 00**	9.93*E* − 01
		±1.87*E* − 02		±6.23*E* − 02	**3.65*E* − 06**	±**1.41*E* − 02**	**7.74*E* − 07**
*f*_9_	5	4.50*E* + 00	2.22*E* + 01	**3.68*E* + 00**	**2.01*E* + 01**	4.74*E* + 00	2.13*E* + 01
		1.28*E* + 01	1.34*E* + 01	1.15*E* + 01	1.15*E* + 01	**1.13*E* + 01**	**1.14*E* + 01**
		±3.52*E* + 00		±**3.40*E* + 00**	**2.35*E* − 04**	±3.46*E* + 00	**5.41*E* − 05**
	10	**2.44*E* + 01**	5.91*E* + 01	2.61*E* + 01	5.87*E* + 01	2.61*E* + 01	**5.60*E* + 01**
		4.44*E* + 01	4.40*E* + 01	4.31*E* + 01	**4.22*E* + 01**	**4.22*E* + 01**	4.24*E* + 01
		±6.68*E* + 00		±**6.01*E* + 00**	**1.93*E* − 02**	±7.00*E* + 00	6.44*E* − 02
*f*_10_	5	—	—	—	—	**6.18*E* + 01**	**9.74*E* + 02**
		—	—	—	—	**5.93*E* + 02**	**5.75*E* + 02**
		—		—	—	±**1.78*E* + 02**	**2.82*E* − 39**
	10	**5.69*E* + 02**	**2.15*E* + 03**	5.93*E* + 02	2.23*E* + 03	7.63*E* + 02	2.24*E* + 03
		**1.77*E* + 03**	**1.71*E* + 03**	1.78*E* + 03	1.76*E* + 03	1.85*E* + 03	1.79*E* + 03
		±2.62*E* + 02		±2.45*E* + 02	8.94*E* − 01	±**2.35*E* + 02**	9.91*E* − 01
*f*_11_	5	4.10*E* + 01	4.31*E* + 01	**4.06*E* + 01**	**4.22*E* + 01**	4.08*E* + 01	4.25*E* + 01
		4.18*E* + 01	4.18*E* + 01	4.15*E* + 01	4.15*E* + 01	**4.14*E* + 01**	**4.14*E* + 01**
		±4.15*E* − 01		±3.47*E* − 01	**9.67*E* − 08**	±**3.11*E* − 01**	**2.23*E* − 10**
	10	1.83*E* + 02	1.87*E* + 02	1.83*E* + 02	1.86*E* + 02	**1.83*E* + 02**	**1.86*E* + 02**
		1.85*E* + 02	1.85*E* + 02	1.85*E* + 02	1.85*E* + 02	**1.85*E* + 02**	**1.85*E* + 02**
		±6.42*E* − 01		±**6.27*E* − 01**	**2.70*E* − 06**	±6.55*E* − 01	**2.38*E* − 08**
*f*_12_	5	3.70*E* + 00	9.50*E* + 00	**2.80*E* + 00**	**8.91*E* + 00**	2.88*E* + 00	9.80*E* + 00
		6.69*E* + 00	6.82*E* + 00	**5.68*E* + 00**	**5.66*E* + 00**	5.84*E* + 00	5.87*E* + 00
		±1.46*E* + 00		±1.30*E* + 00	**1.19*E* − 07**	±**1.23*E* + 00**	**2.67*E* − 06**
	10	5.99*E* + 00	1.30*E* + 01	6.06*E* + 00	**1.13*E* + 01**	**5.01*E* + 00**	1.24*E* + 01
		1.00*E* + 01	9.84*E* + 00	**8.95*E* + 00**	9.13*E* + 00	9.08*E* + 00	**9.12*E* + 00**
		±1.23*E* + 00		±**1.12*E* + 00**	**4.53*E* − 06**	±1.39*E* + 00	**7.36*E* − 05**
*f*_13_	5	2.97*E* − 01	9.57*E* − 01	**1.51*E* − 01**	8.28*E* − 01	1.60*E* − 01	**7.97*E* − 01**
		5.41*E* − 01	5.59*E* − 01	5.08*E* − 01	4.92*E* − 01	**4.77*E* − 01**	**4.85*E* − 01**
		±**1.27*E* − 01**		±1.44*E* − 01	**1.16*E* − 03**	±1.36*E* − 01	**9.77*E* − 05**
	10	8.82*E* − 01	1.28*E* + 00	7.98*E* − 01	1.24*E* + 00	**6.04*E* − 01**	**1.23*E* + 00**
		1.11*E* + 00	1.11*E* + 00	1.08*E* + 00	1.08*E* + 00	**1.07*E* + 00**	**1.06*E* + 00**
		±**7.04*E* − 02**		±7.46*E* − 02	**1.14*E* − 03**	±9.55*E* − 02	**1.91*E* − 05**
*f*_14_	5	**3.09*E* − 01**	2.52*E* + 00	4.48*E* − 01	**2.03*E* + 00**	3.56*E* − 01	2.63*E* + 00
		1.34*E* + 00	1.37*E* + 00	1.20*E* + 00	**1.18*E* + 00**	**1.20*E* + 00**	1.19*E* + 00
		±3.93*E* − 01		±**3.52*E* − 01**	**3.88*E* − 04**	±3.59*E* − 01	**2.14*E* − 04**
	10	1.73*E* + 00	4.20*E* + 00	1.80*E* + 00	4.22*E* + 00	**1.58*E* + 00**	**3.68*E* + 00**
		2.96*E* + 00	2.97*E* + 00	2.72*E* + 00	2.71*E* + 00	**2.71*E* + 00**	**2.66*E* + 00**
		±4.74*E* − 01		±4.77*E* − 01	**6.94*E* − 05**	±**4.66*E* − 01**	**5.25*E* − 06**
*f*_15_	5	5.04*E* − 02	1.55*E* − 01	4.53*E* − 02	1.21*E* − 01	**4.44*E* − 02**	**1.08*E* − 01**
		7.84*E* − 02	8.18*E* − 02	7.22*E* − 02	7.21*E* − 02	**6.74*E* − 02**	**6.88*E* − 02**
		±1.99*E* − 02		±1.59*E* − 02	**2.67*E* − 04**	±**1.44*E* − 02**	**5.03*E* − 07**
	10	1.86*E* − 03	1.27*E* − 02	**1.17*E* − 03**	**1.07*E* − 02**	1.26*E* − 03	1.12*E* − 02
		5.75*E* − 03	5.66*E* − 03	5.28*E* − 03	5.43*E* − 03	**4.93*E* − 03**	**5.12*E* − 03**
		±2.04*E* − 03		±2.03*E* − 03	2.58*E* − 01	±**1.95*E* − 03**	**2.84*E* − 02**

		SPX		SH-SPX_random		SH-SPX_sequential	
*f*_1_	5	2.73*E* − 01	2.23*E* + 00	**1.71*E* − 01**	**1.48*E* + 00**	1.83*E* − 01	2.99*E* + 00
		9.30*E* − 01	9.76*E* − 01	6.46*E* − 01	7.10*E* − 01	**5.77*E* − 01**	**6.90*E* − 01**
		±4.08*E* − 01		±**2.74*E* − 01**	**3.92*E* − 07**	±4.21*E* − 01	**5.93*E* − 09**
	10	2.11*E* − 01	8.83*E* − 01	1.76*E* − 01	9.73*E* − 01	**1.64*E* − 01**	**8.63*E* − 01**
		4.73*E* − 01	5.03*E* − 01	3.55*E* − 01	3.78*E* − 01	**2.81*E* − 01**	**3.07*E* − 01**
		±1.29*E* − 01		±1.26*E* − 01	**4.24*E* − 13**	±**1.22*E* − 01**	**3.26*E* − 21**
*f*_2_	5	3.00*E* − 03	6.10*E* − 02	1.09*E* − 03	6.74*E* − 02	**3.67*E* − 04**	**3.57*E* − 02**
		1.54*E* − 02	1.97*E* − 02	7.82*E* − 03	1.11*E* − 02	**4.51*E* − 03**	**7.39*E* − 03**
		±1.33*E* − 02		±9.97*E* − 03	**2.63*E* − 09**	±**6.87*E* − 03**	**5.70*E* − 17**
	10	9.45*E* − 04	1.11*E* − 02	5.33*E* − 04	7.35*E* − 03	**1.68*E* − 04**	**5.32*E* − 03**
		3.70*E* − 03	4.22*E* − 03	1.64*E* − 03	1.89*E* − 03	**1.08*E* − 03**	**1.30*E* − 03**
		±2.06*E* − 03		±1.07*E* − 03	**1.29*E* − 20**	±**8.56*E* − 04**	**2.16*E* − 27**
*f*_3_	5	4.02*E* − 03	4.15*E* − 01	2.48*E* − 03	**3.84*E* − 01**	**2.41*E* − 03**	4.92*E* − 01
		4.10*E* − 02	5.50*E* − 02	2.21*E* − 02	**3.68*E* − 02**	**1.86*E* − 02**	4.22*E* − 02
		±5.67*E* − 02		±**5.17*E* − 02**	**6.57*E* − 06**	±7.39*E* − 02	**7.37*E* − 07**
	10	5.20*E* − 03	**8.20*E* − 02**	2.42*E* − 03	1.88*E* − 01	**1.66*E* − 03**	1.36*E* − 01
		2.07*E* − 02	2.31*E* − 02	1.31*E* − 02	1.93*E* − 02	**6.25*E* − 03**	**1.38*E* − 02**
		±**1.23*E* − 02**		±2.19*E* − 02	**1.04*E* − 05**	±2.30*E* − 02	**4.27*E* − 17**
*f*_4_	5	5.72*E* + 00	1.10*E* + 03	**2.49*E* + 00**	**6.26*E* + 02**	2.50*E* + 00	2.62*E* + 03
		2.12*E* + 01	**5.79*E* + 01**	1.80*E* + 01	6.39*E* + 01	**1.27*E* + 01**	7.01*E* + 01
		±1.29*E* + 02		±**1.18*E* + 02**	1.74*E* − 01	±2.77*E* + 02	**1.89*E* − 05**
	10	1.04*E* + 01	**8.49*E* + 01**	8.69*E* + 00	2.67*E* + 02	**7.58*E* + 00**	3.46*E* + 02
		1.64*E* + 01	**1.88*E* + 01**	1.19*E* + 01	2.13*E* + 01	**1.06*E* + 01**	1.95*E* + 01
		±**9.64*E* + 00**		±3.56*E* + 01	**6.19*E* − 11**	±3.77*E* + 01	**3.70*E* − 14**
*f*_5_	5	3.63*E* − 01	3.58*E* + 01	1.59*E* − 01	1.27*E* + 02	**7.85*E* − 02**	**3.06*E* + 01**
		2.12*E* + 00	**3.44*E* + 00**	1.92*E* + 00	5.01*E* + 00	**1.58*E* + 00**	4.20*E* + 00
		±**4.57*E* + 00**		±1.41*E* + 01	9.05*E* − 02	±6.01*E* + 00	**2.58*E* − 02**
	10	2.80*E* − 01	**6.40*E* + 00**	7.15*E* − 02	6.69*E* + 00	**4.67*E* − 02**	1.13*E* + 01
		9.01*E* − 01	1.20*E* + 00	5.28*E* − 01	**8.65*E* − 01**	**5.03*E* − 01**	1.19*E* + 00
		±1.04*E* + 00		±**9.14*E* − 01**	**6.06*E* − 07**	±1.79*E* + 00	**9.06*E* − 06**
*f*_6_	5	1.46*E* + 00	2.49*E* + 01	5.51*E* − 01	**1.84*E* + 01**	**1.93*E* − 01**	1.90*E* + 01
		5.66*E* + 00	6.28*E* + 00	3.13*E* + 00	**3.72*E* + 00**	**2.71*E* + 00**	4.45*E* + 00
		±3.41*E* + 00		±**2.47*E* + 00**	**1.05*E* − 15**	±4.19*E* + 00	**2.78*E* − 10**
	10	2.16*E* + 00	1.11*E* + 01	1.42*E* + 00	3.24*E* + 01	**9.44*E* − 01**	**8.43*E* + 00**
		4.13*E* + 00	4.37*E* + 00	2.65*E* + 00	3.26*E* + 00	**2.03*E* + 00**	**2.42*E* + 00**
		±1.35*E* + 00		±3.25*E* + 00	**1.25*E* − 17**	±**1.32*E* + 00**	**1.58*E* − 21**
*f*_7_	5	5.87*E* − 03	**1.12*E* + 00**	4.99*E* − 03	1.34*E* + 00	**3.09*E* − 03**	3.30*E* + 00
		8.86*E* − 02	1.65*E* − 01	**5.66*E* − 02**	**1.38*E* − 01**	8.61*E* − 02	2.95*E* − 01
		±**1.96*E* − 01**		±2.15*E* − 01	**3.69*E* − 03**	±5.54*E* − 01	1.90*E* − 01
	10	1.36*E* − 02	**1.19*E* + 00**	4.93*E* − 03	1.22*E* + 00	**4.72*E* − 03**	1.86*E* + 00
		6.32*E* − 02	1.24*E* − 01	**2.42*E* − 02**	**7.25*E* − 02**	3.80*E* − 02	1.35*E* − 01
		±1.66*E* − 01		±**1.55*E* − 01**	**7.46*E* − 09**	±2.49*E* − 01	**2.72*E* − 03**
*f*_8_	5	—	—	—	—	—	—
		—	—	—	—	—	—
		—		—	—	—	—
	10	3.58*E* − 03	**1.92*E* − 01**	1.20*E* − 03	9.98*E* − 01	**9.28*E* − 04**	9.98*E* − 01
		1.73*E* − 02	**2.54*E* − 02**	**6.69*E* − 03**	5.27*E* − 02	8.29*E* − 03	6.27*E* − 02
		±**2.82*E* − 02**		±1.88*E* − 01	**1.08*E* − 10**	±1.96*E* − 01	**9.22*E* − 05**
*f*_9_	5	2.42*E* + 00	2.32*E* + 01	9.43*E* − 01	2.03*E* + 01	**6.68*E* − 01**	**2.03*E* + 01**
		1.12*E* + 01	1.18*E* + 01	1.02*E* + 01	1.02*E* + 01	**8.62*E* + 00**	**8.86*E* + 00**
		±3.95*E* + 00		±3.89*E* + 00	**4.58*E* − 03**	±**3.45*E* + 00**	**8.84*E* − 09**
	10	1.44*E* + 01	4.54*E* + 01	3.53*E* + 00	3.65*E* + 01	**8.75*E* − 01**	**2.79*E* + 01**
		3.47*E* + 01	3.37*E* + 01	1.52*E* + 01	1.71*E* + 01	**8.14*E* + 00**	**9.53*E* + 00**
		±6.66*E* + 00		±7.99*E* + 00	**6.39*E* − 27**	±**5.97*E* + 00**	**1.87*E* − 33**
*f*_10_	5	—	—	—	—	—	—
		—	—	—	—	—	—
		—		—	—	—	—
	10	—	—	—	—	**9.16*E* + 00**	**2.86*E* + 03**
		—	—	—	—	**1.88*E* + 03**	**1.83*E* + 03**
		—		—	—	±**4.94*E* + 02**	**2.82*E* − 39**
*f*_11_	5	4.04*E* + 01	4.13*E* + 01	4.03*E* + 01	4.15*E* + 01	**4.02*E* + 01**	**4.11*E* + 01**
		4.07*E* + 01	4.07*E* + 01	4.06*E* + 01	4.06*E* + 01	**4.05*E* + 01**	**4.05*E* + 01**
		±1.90*E* − 01		±**1.60*E* − 01**	**4.35*E* − 10**	±1.65*E* − 01	**8.70*E* − 15**
	10	1.81*E* + 02	1.81*E* + 02	1.80*E* + 02	**1.81*E* + 02**	**1.80*E* + 02**	1.81*E* + 02
		1.81*E* + 02	1.81*E* + 02	1.81*E* + 02	1.81*E* + 02	**1.81*E* + 02**	**1.81*E* + 02**
		±1.29*E* − 01		±**1.08*E* − 01**	**4.36*E* − 18**	±1.28*E* − 01	**1.14*E* − 25**
*f*_12_	5	4.05*E* − 01	3.28*E* + 00	2.13*E* − 01	3.14*E* + 00	**1.97*E* − 01**	**2.65*E* + 00**
		1.87*E* + 00	1.84*E* + 00	1.45*E* + 00	1.49*E* + 00	**1.12*E* + 00**	**1.23*E* + 00**
		±5.88*E* − 01		±6.29*E* − 01	**4.46*E* − 05**	±**5.57*E* − 01**	**1.65*E* − 11**
	10	2.67*E* − 01	1.39*E* + 00	1.57*E* − 01	2.07*E* + 00	**8.07*E* − 02**	**5.88*E* − 01**
		5.83*E* − 01	6.16*E* − 01	3.50*E* − 01	3.96*E* − 01	**2.36*E* − 01**	**2.45*E* − 01**
		±2.25*E* − 01		±2.25*E* − 01	**2.84*E* − 16**	±**9.53*E* − 02**	**4.47*E* − 30**
*f*_13_	5	1.04*E* − 01	6.17*E* − 01	**4.94*E* − 02**	**5.92*E* − 01**	9.47*E* − 02	5.98*E* − 01
		3.49*E* − 01	3.52*E* − 01	**3.12*E* − 01**	**3.20*E* − 01**	3.31*E* − 01	3.27*E* − 01
		±1.11*E* − 01		±1.12*E* − 01	**2.86*E* − 02**	±**1.09*E* − 01**	7.84*E* − 02
	10	6.96*E* − 02	4.84*E* − 01	1.43*E* − 02	3.90*E* − 01	**1.22*E* − 02**	**2.88*E* − 01**
		1.93*E* − 01	2.08*E* − 01	7.37*E* − 02	9.07*E* − 02	**5.03*E* − 02**	**6.06*E* − 02**
		±1.00*E* − 01		±6.20*E* − 02	**6.30*E* − 20**	±**4.44*E* − 02**	**6.33*E* − 28**
*f*_14_	5	2.00*E* − 01	7.73*E* − 01	**1.21*E* − 01**	**7.01*E* − 01**	2.00*E* − 01	7.10*E* − 01
		4.13*E* − 01	4.24*E* − 01	4.00*E* − 01	3.98*E* − 01	**4.00*E* − 01**	**3.86*E* − 01**
		±1.39*E* − 01		±1.30*E* − 01	1.16*E* − 01	±**1.22*E* − 01**	**3.43*E* − 02**
	10	2.00*E* − 01	7.03*E* − 01	2.00*E* − 01	**5.14*E* − 01**	**2.00*E* − 01**	6.16*E* − 01
		3.65*E* − 01	3.66*E* − 01	3.10*E* − 01	**3.25*E* − 01**	**3.08*E* − 01**	3.35*E* − 01
		±8.08*E* − 02		±**6.94*E* − 02**	**2.20*E* − 04**	±9.77*E* − 02	**1.57*E* − 03**
*f*_15_	5	4.37*E* − 02	1.27*E* − 01	**4.24*E* − 02**	1.32*E* − 01	4.28*E* − 02	**8.59*E* − 02**
		7.25*E* − 02	7.30*E* − 02	5.54*E* − 02	5.82*E* − 02	**5.12*E* − 02**	**5.45*E* − 02**
		±1.63*E* − 02		±1.36*E* − 02	**2.32*E* − 12**	±**9.05*E* − 03**	**5.28*E* − 18**
	10	1.08*E* − 03	9.64*E* − 03	6.29*E* − 04	5.55*E* − 03	**6.01*E* − 04**	**3.29*E* − 03**
		4.23*E* − 03	4.38*E* − 03	1.22*E* − 03	1.52*E* − 03	**8.99*E* − 04**	**1.01*E* − 03**
		±1.89*E* − 03		±8.58*E* − 04	**2.60*E* − 27**	±**4.11*E* − 04**	**2.82*E* − 33**

		UNDX		SH-UNDX_random		SH-UNDX_sequential	
*f*_1_	5	1.20*E* + 00	6.82*E* + 00	8.26*E* − 01	**5.16*E* + 00**	**7.35*E* − 01**	5.54*E* + 00
		2.77*E* + 00	2.83*E* + 00	2.18*E* + 00	2.30*E* + 00	**1.96*E* + 00**	**2.13*E* + 00**
		±1.09*E* + 00		±**8.41*E* − 01**	**1.60*E* − 04**	±8.74*E* − 01	**3.29*E* − 07**
	10	2.04*E* + 00	6.43*E* + 00	**1.36*E* + 00**	5.53*E* + 00	1.71*E* + 00	**5.33*E* + 00**
		4.25*E* + 00	4.25*E* + 00	3.65*E* + 00	3.66*E* + 00	**3.54*E* + 00**	**3.50*E* + 00**
		±1.01*E* + 00		±7.54*E* − 01	**9.57*E* − 06**	±**7.34*E* − 01**	**2.52*E* − 08**
*f*_2_	5	1.30*E* − 02	6.07*E* − 01	1.24*E* − 02	**4.28*E* − 01**	**4.48*E* − 03**	6.26*E* − 01
		1.30*E* − 01	1.60*E* − 01	**6.85*E* − 02**	**8.27*E* − 02**	7.20*E* − 02	8.83*E* − 02
		±1.16*E* − 01		±**6.00*E* − 02**	**7.89*E* − 09**	±8.50*E* − 02	**1.94*E* − 08**
	10	9.42*E* − 02	1.26*E* + 00	7.07*E* − 02	6.85*E* − 01	**4.32*E* − 02**	**6.42*E* − 01**
		4.10*E* − 01	4.74*E* − 01	2.98*E* − 01	3.03*E* − 01	**2.49*E* − 01**	**2.68*E* − 01**
		±2.39*E* − 01		±1.43*E* − 01	**3.09*E* − 08**	±**1.22*E* − 01**	**2.28*E* − 12**
*f*_3_	5	4.93*E* − 02	1.77*E* + 00	**1.51*E* − 02**	1.81*E* + 00	2.90*E* − 02	**8.85*E* − 01**
		3.45*E* − 01	4.71*E* − 01	2.17*E* − 01	2.96*E* − 01	**2.03*E* − 01**	**2.71*E* − 01**
		±3.48*E* − 01		±2.77*E* − 01	**2.17*E* − 06**	±**2.04*E* − 01**	**6.85*E* − 07**
	10	3.27*E* − 01	5.08*E* + 00	**2.47*E* − 01**	6.39*E* + 00	4.47*E* − 01	**4.88*E* + 00**
		2.30*E* + 00	2.30*E* + 00	**1.47*E* + 00**	1.72*E* + 00	1.49*E* + 00	**1.69*E* + 00**
		±9.70*E* − 01		±9.66*E* − 01	**6.77*E* − 07**	±**8.96*E* − 01**	**7.28*E* − 07**
*f*_4_	5	**4.89*E* + 00**	1.54*E* + 03	6.89*E* + 00	1.21*E* + 03	6.88*E* + 00	**1.17*E* + 03**
		1.51*E* + 02	2.41*E* + 02	**8.81*E* + 01**	1.93*E* + 02	1.00*E* + 02	**1.60*E* + 02**
		±2.70*E* + 02		±2.38*E* + 02	**2.12*E* − 02**	±**1.80*E* + 02**	**8.40*E* − 03**
	10	1.04*E* + 02	3.64*E* + 03	**8.64*E* + 01**	**1.54*E* + 03**	9.04*E* + 01	3.07*E* + 03
		6.38*E* + 02	7.67*E* + 02	4.29*E* + 02	4.78*E* + 02	**3.65*E* + 02**	**4.71*E* + 02**
		±5.61*E* + 02		±**2.72*E* + 02**	**5.55*E* − 06**	±4.01*E* + 02	**6.08*E* − 08**
*f*_5_	5	**6.70*E* − 01**	8.64*E* + 01	2.11*E* + 00	2.42*E* + 02	2.50*E* + 00	**6.09*E* + 01**
		2.07*E* + 01	2.40*E* + 01	1.58*E* + 01	2.33*E* + 01	**1.49*E* + 01**	**1.80*E* + 01**
		±1.64*E* + 01		±2.85*E* + 01	**4.19*E* − 02**	±**1.30*E* + 01**	**3.00*E* − 03**
	10	2.25*E* + 01	2.33*E* + 02	2.88*E* + 01	2.05*E* + 02	**1.91*E* + 01**	**2.01*E* + 02**
		1.02*E* + 02	1.04*E* + 02	**7.97*E* + 01**	**8.55*E* + 01**	8.47*E* + 01	8.99*E* + 01
		±4.18*E* + 01		±**3.30*E* + 01**	**2.52*E* − 04**	±3.96*E* + 01	**4.88*E* − 03**
*f*_6_	5	2.16*E* + 00	4.98*E* + 01	3.22*E* + 00	**4.47*E* + 01**	**1.94*E* + 00**	5.04*E* + 01
		1.44*E* + 01	1.62*E* + 01	**9.27*E* + 00**	**1.16*E* + 01**	1.22*E* + 01	1.28*E* + 01
		±9.25*E* + 00		±**6.70*E* + 00**	**3.06*E* − 05**	±7.34*E* + 00	**2.07*E* − 03**
	10	1.54*E* + 01	3.08*E* + 03	1.46*E* + 01	**5.00*E* + 02**	**1.39*E* + 01**	1.19*E* + 03
		5.27*E* + 01	1.50*E* + 02	3.49*E* + 01	**5.30*E* + 01**	**3.08*E* + 01**	5.44*E* + 01
		±3.87*E* + 02		±**7.00*E* + 01**	**1.12*E* − 05**	±1.22*E* + 02	**4.08*E* − 09**
*f*_7_	5	1.00*E* − 01	5.44*E* + 00	**2.71*E* − 02**	**3.31*E* + 00**	2.87*E* − 02	5.63*E* + 00
		8.04*E* − 01	1.16*E* + 00	**5.35*E* − 01**	**8.39*E* − 01**	5.39*E* − 01	9.49*E* − 01
		±1.02*E* + 00		±**7.72*E* − 01**	**3.33*E* − 03**	±9.58*E* − 01	**1.35*E* − 02**
	10	1.84*E* + 00	1.60*E* + 01	2.16*E* + 00	**1.40*E* + 01**	**8.81*E* − 01**	1.48*E* + 01
		6.18*E* + 00	6.60*E* + 00	**5.08*E* + 00**	5.83*E* + 00	5.21*E* + 00	**5.65*E* + 00**
		±2.97*E* + 00		±**2.90*E* + 00**	**1.62*E* − 02**	±3.05*E* + 00	**5.28*E* − 03**
*f*_8_	5	—	—	—	—	—	—
		—	—	—	—	—	—
		—		—	—	—	—
	10	8.00*E* − 01	**1.00*E* + 00**	7.95*E* − 01	1.00*E* + 00	**4.05*E* − 01**	1.00*E* + 00
		1.00*E* + 00	9.92*E* − 01	**9.96*E* − 01**	9.78*E* − 01	9.99*E* − 01	**9.72*E* − 01**
		±**2.60*E* − 02**		±4.13*E* − 02	**6.41*E* − 08**	±8.29*E* − 02	**1.42*E* − 04**
*f*_9_	5	3.55*E* + 00	1.95*E* + 01	3.51*E* + 00	1.96*E* + 01	**1.81*E* + 00**	**1.89*E* + 01**
		1.06*E* + 01	1.10*E* + 01	1.10*E* + 01	1.09*E* + 01	**1.00*E* + 01**	**1.01*E* + 01**
		±3.58*E* + 00		±**3.30*E* + 00**	4.28*E* − 01	±3.50*E* + 00	**4.51*E* − 02**
	10	1.74*E* + 01	4.84*E* + 01	1.95*E* + 01	**4.38*E* + 01**	**1.70*E* + 01**	4.69*E* + 01
		3.64*E* + 01	3.64*E* + 01	3.45*E* + 01	**3.40*E* + 01**	**3.45*E* + 01**	3.43*E* + 01
		±6.45*E* + 00		±**5.52*E* + 00**	**1.98*E* − 03**	±6.07*E* + 00	**7.45*E* − 03**
*f*_10_	5	—	—	—	—	**6.48*E* + 01**	**1.16*E* + 03**
		—	—	—	—	**7.66*E* + 02**	**7.24*E* + 02**
		—		—	—	±**2.55*E* + 02**	**2.82*E* − 39**
	10	—	—	—	—	**5.25*E* + 02**	**2.48*E* + 03**
		—	—	—	—	**1.70*E* + 03**	**1.60*E* + 03**
		—		—	—	±**4.68*E* + 02**	**2.82*E* − 39**
*f*_11_	5	4.06*E* + 01	4.22*E* + 01	4.06*E* + 01	4.19*E* + 01	**4.05*E* + 01**	**4.19*E* + 01**
		4.12*E* + 01	4.13*E* + 01	**4.11*E* + 01**	**4.11*E* + 01**	4.11*E* + 01	4.11*E* + 01
		±3.29*E* − 01		±2.95*E* − 01	**2.24*E* − 04**	±**2.75*E* − 01**	**4.55*E* − 04**
	10	**1.82*E* + 02**	1.84*E* + 02	1.82*E* + 02	**1.84*E* + 02**	1.82*E* + 02	1.84*E* + 02
		1.83*E* + 02	1.83*E* + 02	1.83*E* + 02	1.83*E* + 02	**1.83*E* + 02**	**1.83*E* + 02**
		±4.72*E* − 01		±**4.03*E* − 01**	**5.09*E* − 07**	±4.09*E* − 01	**2.62*E* − 08**
*f*_12_	5	2.04*E* + 00	5.83*E* + 00	1.69*E* + 00	**4.95*E* + 00**	**1.27*E* + 00**	5.92*E* + 00
		3.89*E* + 00	3.81*E* + 00	**3.23*E* + 00**	**3.24*E* + 00**	3.30*E* + 00	3.26*E* + 00
		±7.79*E* − 01		±**6.47*E* − 01**	**1.07*E* − 07**	±8.19*E* − 01	**1.42*E* − 06**
	10	3.04*E* + 00	6.14*E* + 00	2.47*E* + 00	5.22*E* + 00	**1.85*E* + 00**	**5.06*E* + 00**
		4.32*E* + 00	4.38*E* + 00	3.87*E* + 00	3.85*E* + 00	**3.81*E* + 00**	**3.81*E* + 00**
		±6.26*E* − 01		±**5.59*E* − 01**	**2.45*E* − 08**	±5.87*E* − 01	**4.85*E* − 09**
*f*_13_	5	1.54*E* − 01	6.60*E* − 01	**1.07*E* − 01**	**5.25*E* − 01**	1.55*E* − 01	6.38*E* − 01
		3.74*E* − 01	3.81*E* − 01	3.73*E* − 01	3.64*E* − 01	**3.39*E* − 01**	**3.55*E* − 01**
		±1.06*E* − 01		±**9.63*E* − 02**	2.13*E* − 01	±1.01*E* − 01	**3.81*E* − 02**
	10	4.11*E* − 01	9.76*E* − 01	**4.01*E* − 01**	9.11*E* − 01	4.77*E* − 01	**8.96*E* − 01**
		8.37*E* − 01	8.09*E* − 01	7.95*E* − 01	7.73*E* − 01	**7.59*E* − 01**	**7.47*E* − 01**
		±1.06*E* − 01		±**9.53*E* − 02**	**9.46*E* − 04**	±1.06*E* − 01	**2.73*E* − 06**
*f*_14_	5	2.01*E* − 01	1.13*E* + 00	2.15*E* − 01	**9.63*E* − 01**	**1.46*E* − 01**	1.03*E* + 00
		7.05*E* − 01	7.00*E* − 01	5.80*E* − 01	5.79*E* − 01	**5.41*E* − 01**	**5.68*E* − 01**
		±1.94*E* − 01		±**1.72*E* − 01**	**6.01*E* − 06**	±1.83*E* − 01	**1.40*E* − 06**
	10	6.09*E* − 01	1.69*E* + 00	5.27*E* − 01	1.40*E* + 00	**5.00*E* − 01**	**1.31*E* + 00**
		1.00*E* + 00	1.04*E* + 00	9.04*E* − 01	9.09*E* − 01	**8.95*E* − 01**	**8.80*E* − 01**
		±2.16*E* − 01		±1.72*E* − 01	**1.79*E* − 05**	±**1.58*E* − 01**	**9.29*E* − 08**
*f*_15_	5	**4.42*E* − 02**	**1.08*E* − 01**	4.62*E* − 02	1.09*E* − 01	4.67*E* − 02	1.13*E* − 01
		7.04*E* − 02	7.25*E* − 02	6.97*E* − 02	7.06*E* − 02	**6.76*E* − 02**	**6.93*E* − 02**
		±1.45*E* − 02		±**1.34*E* − 02**	1.99*E* − 01	±1.39*E* − 02	6.66*E* − 02
	10	1.44*E* − 03	**7.63*E* − 03**	1.89*E* − 03	8.04*E* − 03	**1.35*E* − 03**	8.53*E* − 03
		4.31*E* − 03	4.33*E* − 03	**4.02*E* − 03**	4.24*E* − 03	4.12*E* − 03	**4.23*E* − 03**
		±**1.30*E* − 03**		±1.31*E* − 03	2.75*E* − 01	±1.38*E* − 03	2.68*E* − 01

The best results in each row are emphasized in bold. The emphasized *p* values in bold indicate that the Mann–Whitney *U* test with the significance level *p*=0.05 shows significance against the result without SHX. “—” represents invalid solutions (trapped by local optimum or out of parameter range

## Data Availability

The test data used to support the findings of this study are included within the article.
